# Resting state electroencephalographic rhythms are affected by immediately preceding memory demands in cognitively unimpaired elderly and patients with mild cognitive impairment

**DOI:** 10.3389/fnagi.2022.907130

**Published:** 2022-08-05

**Authors:** Alba Fernández, Giuseppe Noce, Claudio Del Percio, Diego Pinal, Fernando Díaz, Cristina Lojo-Seoane, Montserrat Zurrón, Claudio Babiloni

**Affiliations:** ^1^Departamento de Psicoloxía Clínica e Psicobioloxía, Facultade de Psicoloxía, Universidade de Santiago de Compostela, Santiago de Compostela, Spain; ^2^IRCCS Synlab SDN, Naples, Italy; ^3^Department of Physiology and Pharmacology “V. Erspamer”, Sapienza University of Rome, Rome, Italy; ^4^Psychological Neuroscience Lab, Escola de Psicologia, Universidade do Minho, Braga, Portugal; ^5^Departamento de Psicoloxía Evolutiva e da Educación, Facultade de Psicoloxía, Universidade de Santiago de Compostela, Santiago de Compostela, Spain; ^6^San Raffaele Cassino, Cassino, Italy

**Keywords:** resting state EEG power, alpha oscillations, cortical arousal, mild cognitive impairment, cognitive engagement, memory, aging

## Abstract

Experiments on event-related electroencephalographic oscillations in aged people typically include blocks of cognitive tasks with a few minutes of interval between them. The present exploratory study tested the effect of being engaged on cognitive tasks over the resting state cortical arousal after task completion, and whether it differs according to the level of the participant’s cognitive decline. To investigate this issue, we used a local database including data in 30 healthy cognitively unimpaired (CU) persons and 40 matched patients with amnestic mild cognitive impairment (aMCI). They had been involved in 2 memory tasks for about 40 min and underwent resting-state electroencephalographic (rsEEG) recording after 5 min from the task end. eLORETA freeware estimated rsEEG alpha source activity as an index of general cortical arousal. In the CU but not aMCI group, there was a negative correlation between memory tasks performance and posterior rsEEG alpha source activity. The better the memory tasks performance, the lower the posterior alpha activity (i.e., higher cortical arousal). There was also a negative correlation between neuropsychological test scores of global cognitive status and alpha source activity. These results suggest that engagement in memory tasks may perturb background brain arousal for more than 5 min after the tasks end, and that this effect are dependent on participants global cognitive status. Future studies in CU and aMCI groups may cross-validate and extend these results with experiments including (1) rsEEG recordings before memory tasks and (2) post-tasks rsEEG recordings after 5, 15, and 30 min.

## Introduction

Rhythmic oscillations at multiple frequencies of brain neural activities underpin cognitive processes and can be measured by electroencephalographic (EEG) techniques (David et al., 2006). Those oscillations are related to neural communications (Başar and Düzgün, 2016) *via* the modulation of neuronal spiking and the synchronization of nodes within cortical networks (Zhang et al., 2018). They are usually classified into the following canonical frequency bands: delta (1–4 Hz), theta (4–8 Hz), alpha (8–12 Hz), beta (13–30 Hz), and gamma (>30 Hz) (Cole and Voytek, 2017).

Among them, alpha oscillations are prominent in parietal-occipital areas and are extremely important for human higher cognitive functions (Chen et al., 2008). During working memory (WM) tasks, a reduction in amplitude of EEG alpha activity may represent a gating neurophysiological mechanism that would facilitate cognitive information processing in task-relevant cortical areas (de Vries et al., 2020) while an increase in that amplitude would inhibit the information processing in task-irrelevant cortical areas (Klimesch, 1999; Jensen and Mazaheri, 2010; van Ede, 2018). For example, an increase in alpha activity in visual posterior cortical areas would inhibit (task-irrelevant) visual information processing during maintenance of auditory stimuli in WM [(van Ede, 2018), for a review see Pavlov et al. (2020)]. Furthermore, a decrease in alpha activity in visual posterior cortical areas would facilitate (task-relevant) maintenance of visual stimuli during visual WM tasks (Ester et al., 2018; de Vries et al., 2020) or its current use (de Vries et al., 2017, 2018).

Electroencephalographic alpha oscillations are also relevant during the retrieval of episodic memory (EM) information. As well as with WM, alpha activity during EM has also been interpreted as a mechanism working in a similar fashion by which a reduction of alpha power would facilitate the cognitive processing (Jensen and Mazaheri, 2010; Klimesch, 2012). This reduction in alpha activity shows a distinct topography depending on the specific information to be remembered (Burgess and Gruzelier, 2000; Hanslmayr et al., 2012), and provides favorable conditions for the appearance of complex neuronal patterns (Cohen and Kohn, 2011; Cui et al., 2016; Hanslmayr et al., 2016; Clayton et al., 2018). Summarizing, increased EEG alpha activity would reflect the inhibition in thalamocortical and cortical neural networks representing irrelevant sensory inputs and stored information, while reduced alpha activity would reflect the reactivation of the relevant ones during task-related sensory and memory information processing (Jensen and Mazaheri, 2010; Klimesch, 2012).

Electroencephalographic alpha oscillations recorded during resting state are also very informative about human higher cognitive functions. Dominant posterior alpha oscillations during resting-state eyes-closed EEG (rsEEG) recordings are associated with healthy brain functions by regulating vigilance in wakefulness, hence, being an index of background cortical arousal (Ikezawa et al., 2011; Babiloni et al., 2020a). Alpha oscillations are also related to neuropsychological indexes of WM capacity, as measured by Wechsler Adult Intelligence Scale (WAIS-IV) and the Wechsler Memory Scale (WMS-IV) scores (Oswald et al., 2017), and to global cognition, as measured by the Mini Mental State Examination (MMSE) scale (Babiloni et al., 2015, 2017b,^2018^). Furthermore, rsEEG alpha rhythms in parietal-occipital regions show reduced amplitude in relation to the decline in cognitive functions associated with healthy (Klimesch, 1997; Polich, 1997; Babiloni et al., 2006) and pathological aging, including neurodegenerative dementing diseases [(Jafari et al., 2020), for a review see Lejko et al. (2020)]. Several studies have found reduced posterior rsEEG alpha activity in elderly adults with amnestic mild cognitive impairment (aMCI) as compared to cognitively unimpaired (CU) seniors (Roh et al., 2011; Babiloni et al., 2017b,^2018^, ^2021^; Lizio et al., 2018). In the same line, reduced rsEEG alpha activity was found in patients with Alzheimer’s disease dementia (ADD) as compared to aMCI patients (Babiloni et al., 2009, 2013) and even in aMCI patients that later progressed to ADD as compared with stable aMCI (Huang et al., 2000; Luckhaus et al., 2008; Gouw et al., 2017). Furthermore, abnormalities in rsEEG alpha activity were related to cortical neurodegeneration in the volume and density of cortical gray matter as well as with declines in cognitive test scores in patients with aMCI and ADD (Babiloni et al., 2013, 2015).

Furthermore, as a marker of cortical arousal, alpha oscillations are sensitive to mental fatigue. Thus, research analyzing the effects of mental fatigue on EEG signals, has shown that there are changes in participants’ alpha activity from the start to the end of a sequence of cognitive tasks. These changes are further associated with the amount of cognitive effort involved in those tasks (for reviews see, Borghini et al., 2014; Tran et al., 2020), and may last for several minutes after the end of those cognitive tasks (see Jacquet et al., 2021a,b).

Keeping in mind the above data and considerations about the role that alpha activity plays in neurocognitive functioning, in the present work we want to shed light on whether the neurofunctional characteristics associated with alpha activity can be affected by performing a sequence of cognitive tasks across an EEG recording session with several tasks or blocks. Exploring such possibility is especially relevant since most typical experiments on event-related EEG alpha oscillations in aged people usually include several blocks of memory tasks with a few minutes of interval between them (e.g., Van der Hiele et al., 2007a; van der Hiele et al., 2007b; Deiber et al., 2011; Prieto del Val et al., 2016; Nguyen et al., 2017; Fraga et al., 2018; Goodman et al., 2019). Therefore, the present work stems from the need of an exploration of the effects of cognitive engagement in memory tasks on the neurophysiological mechanisms regulating EEG alpha rhythms during a subsequent rest period in healthy elderly adults and matched adults with MCI. Therefore, the present exploratory study tested whether background cortical arousal a few minutes after a cognitive task, as indexed by alpha activity, show similar properties and relationships with cognition as those observed in traditional studies of rsEEG alpha oscillations. Further, we tested if the participant’s cognitive status (i.e., CU and aMCI) might influence background cortical arousal after a cognitive task.

To that end, we used archived data in CU persons and matched aMCI patients available in our Consortium (PDWAVES Consortium). These participants had been involved in two memory tasks for about 40 min, and, then, underwent a rsEEG recording 5 min after the end of those tasks. The lack of a pre-task recording of rsEEG activity did not allow to test the differences in rsEEG alpha activity before and after the memory tasks. However, the design allowed the following predictions: (a) that the participants’ cortical arousal 5 min after the end of the memory tasks may be related to the degree of cognitive effort invested in the memory task (as reflected by memory performance and neuropsychological scores); and (b) that it may further differ according to the individual cognitive status. A confirmation of these hypotheses would encourage investments for new experiments in CU and aMCI seniors with rsEEG recordings before and after memory tasks, and with different intervals between the end of the memory tasks and the post-task rsEEG recordings.

## Material and methods

### Participants

Participants selected for this study were part of a larger sample that was previously recruited by the University of Santiago de Compostela as part of the longitudinal Compostela Aging Study (CompAS). They were referred by their primary care health centers in Santiago de Compostela (Galicia, Spain). All experiments were performed with the informed and written consent of each participant or caregiver, who also consented to the archive of their data for retrospective studies. The study was approved by the Galician Clinical Research Ethics Committee (Xunta de Galicia, Spain) in line with the 1964 Declaration of Helsinki ethical standards (Lynöe et al., 1991).

Archived data of 30 CU seniors and 40 patients with aMCI carefully matched for age, gender and education were used to test the study hypotheses. This data comprised neuropsychological, behavioral and rsEEG signals. The most relevant demographic and clinical variables of each group, as well as the presence or lack of significant differences between the groups, are reported in Table 1.

**TABLE 1:** Mean values (±SD) of the demographic and clinical data in the groups of CU and aMCI seniors, and *p*-value of their statistical comparisons.

	CU	aMCI	Statistical analysis
**Demographic variables**			
*n*	30	40	
Age	67.67 (±8.07)	69.23 (±8.97)	*t*-value = 0.75 (*p* = 0.456) (*t*-test for independent samples)
Education (years)	11.07 (±3.19)	9.95 (±4.33)	*t*-value = −1.19 (*p* = 0.238) (*t*-test for independent samples)
Gender (F:M)	24:6	23:17	*p* = 0.071 (Fisher exact test)
**Clinical markers**			
Geriatric depression scale	1.9 (±3.07)	3.08 (±3.1)	*t*-value = 1.58 (*p* = 0.12) (*t*-test for independent samples)
Global deterioration scale (GDS)	1	3	n/a

F/M, females/males; n/a, not applicable. Global Deterioration Scale: 1 = no cognitive decline; 3 = mild cognitive decline (mild cognitive impairment).

The exclusion criteria to take part in the study were the following: (1) being diagnosed with depression or other psychiatric disorder according to the criteria of the Diagnostic and Statistical Manual of Mental Disorders, Fifth Edition (American Psychiatric Association, 2013); (2) being diagnosed with a neurological disease, including probable ADD or other types of dementia according to the DSM-5 and the National Institute of Neurological and Communicative Disorders and Stroke and the Alzheimer’s Disease and Related Disorders Association (NINCDS-ADRDA) criteria; (3) history of brain damage or brain surgery; (4) previous chemotherapy; (5) being diagnosed with diabetes type-II; (6) sensory or motor disturbances; and (7) consumption or substance abuse/dependence of substances that might affect normal performance of the tasks (i.e., alcohol or drugs).

Regarding their general health status, 14 participants reported taking diabetes medication (13 aMCI, 1 CU); 35 participants reported being treated for hypertension (22 aMCI, 13 CU); 23 participants were taking medication for hypercholesterolemia (17 aMCI, 6 CU); and 1 aMCI participant was taking anti-inflammatory medication. 27 participants reported not being on any of these medications.

Regarding psychoactive medication, two participants (aMCI) reported taking antidepressants and three participants reported taking anxiolytics (two aMCI and one CU). No participant reported taking antipsychotics or dementia medication.

All participants had normal or corrected–to-normal vision. 66 participants were right-handed (hence, four left–handed) as evaluated by the Edinburgh Handedness Inventory (Oldfield, 1971).

### Amnestic mild cognitive impairment diagnostic criteria

All participants underwent a clinical, neurological, and neuropsychological examination before the experimental session. These examinations were conducted by general practitioners, as well as neurologists and neuropsychologists specialized in aging and dementia, respectively.

For the neuropsychological evaluation, the following neuropsychological tests with Spanish norms for age and education were administered to all participants in order to identify those suffering from aMCI: (1) global cognitive status was tested by Spanish version (Lobo et al., 1999) of the MMSE (Folstein et al., 1975) and the Cambridge Cognitive Assessment-Revised battery (CAMCOG-R) [(Roth et al., 1986); Spanish version, (López-Pousa, 2003); Spanish norms (Pereiro Rozas et al., 2015)]; (2) Attention was evaluated with the Trail Making Test A (TMT-A) [(Reynolds, 2002); Spanish norms NEURONORMA (Peña-Casanova et al., 2009c)] and the Attention and Calculation subscale of the CAMCOG-R; (3) Executive functions were assessed with the Trail Making Test B (TMT-B) [(Reynolds, 2002); Spanish norms NEURONORMA (Peña-Casanova et al., 2009c)], Phonological verbal fluency for the letter p [(Lezak et al., 2004); Spanish norms NEURONORMA (Peña-Casanova et al., 2009b)], and the Executive Function CAMCOG-R subscale; (4) Memory was evaluated with the Spanish version (Benedet and Alejandre, 1998) of the California Verbal Learning Test (CVLT) (Delis et al., 1987) and the Memory CAMCOG-R subscale; and (5) Language was evaluated with the Spanish version of the Boston Naming Test (BNT) [(Williams et al., 1989); Spanish norms NEURONORMA (Peña-Casanova et al., 2009a)], semantic verbal fluency (animals) (Lezak et al., 2004), and the Language CAMCOG-R subscale.

Mild cognitive impairment status was diagnosed according to Petersen’s criteria (Petersen, 2004; Albert et al., 2011): (a) evidence of concern, corroborated by an informant, about a change in cognition, relative to the previous level; (b) evidence of poorer performance in one or more cognitive domains that is greater than expected for the patient’s age and educational background; this criteria was considered fulfilled when scoring within the range from 1.00 to 1.99 standard deviations below the norm by age and education (American Psychiatric Association, 2013); (c) preservation of independence in functional abilities; and (d) non-fulfillment of diagnostic criteria for dementia (NINCDS-ADRDA and DSM-5 criteria). Participants included in this study were diagnosed as having aMCI when their memory was among the cognitive domains affected by the impairment, as reflected by their scores in the Spanish version of the CVLT and the Memory CAMCOG-R subscale.

### Neuropsychological data

For this exploratory study, and in order to reduce the number of statistical tests performed, the following scores were selected (see Table 2): (i) MMSE score and total score in the CAMCOG-R battery in order to have measures of global cognitive function. (ii) An index derived from the Trail Making Test (i.e., the difference score: form B–form A) for a measure of executive control processes. Memory scores from the psychological tests were not selected for further analyses since performance in a WM and an EM tasks were also employed in the study analyses (see below for details).

**TABLE 2:** Mean values (±SD) of the neuropsychological scores of the tests used in the present study, in the groups of CU and aMCI seniors.

	CU	aMCI
**Neuropsychological scores**		
MMSE score	28.67 (±1.63)	26.8 (±2.41)
CAMCOG battery total score	95.47 (±5.66)	85.15 (±10.12)
Trail making test (Form A)	45.03 (±15.61)	76.1 (±54.69)
Trail making test (Form B)	108.13 (±55.83)	204.03 (±134.59)
Trail making test Derived Index (B–A)	63.1 (±44.04)	130.16 (±90.86)

### Cognitive tasks

Participants performed two memory tasks prior to the rsEEG recording: a WM task (duration of 15 min) and an EM task with a study phase (15 min), a delay period (20 min) and a phase of delayed recognition (5 min). The WM task was performed during the delay period of the EM task.

#### Working memory task

The WM task was a delayed matching-to-sample (DMS) visuospatial task. This task contained 103 trials. Each trial began with a warning sound followed by the appearance of a sample stimulus that participants had been instructed to memorize. This was followed by a maintenance period randomly ranging between 3250 and 3750 ms, after which a probe stimulus appeared on screen, upon which participants had to decide whether it matched the sample stimulus or not by pressing a different button for each condition. Trials were separated by an intertrial interval ranging between 900 and 1100 ms.

The sample stimulus consisted of three domino tiles and participants were instructed to memorize the number and location of the dots within each tile; while the probe stimulus was formed as well by three domino tiles that could either match the sample stimulus or have one of the dots in a different location for one of the tiles.

Stimulus presentation was semi-randomized, so that no more than three “match” or “non-match” consecutive trials would appear. 50% of trials were “match” and 50% were “non-match.”

Performance in the task was measured with the following variables: percentage of correct responses (i.e., answering match to a match trial or answering non-match to a non-match trial); and reaction time separately for both, correct and error trials (i.e., answering match to a non-match trial or answering non-match to a match trial) (Table 3).

**TABLE 3:** Mean values (±SD) for the performance in the delayed recognition phase of episodic memory task and in the working memory task for CU seniors and patients with aMCI.

	CU	aMCI
**Episodic memory task**		
Correct responses (%)	90.3 (±5.6)	77.4 (±16.2)
Mean reaction time to correct words (ms)	864 (±139)	1126 (±318)
**Working memory task**		
Correct responses (%)	67.6 (±10.6)	55 (±17.3)
Mean reaction time to correct responses (ms)	1288 (±238)	1284 (±270)
Mean reaction time to errors (ms)	1443 (±318)	1457 (±458)

#### Episodic memory task

The EM task was an old/new recognition task with an encoding phase in which participants were instructed to memorize a list of 30 words. The list was repeated three times, with a different random order of its elements each time, for a total duration of 15 min for the encoding phase. There was also a delayed recognition phase that was performed 20 min later. In this delayed recognition phase the participants were sequentially presented 50 “new” words that had not been presented before, and the 30 “old” words that were memorized in the encoding phase. Only the old words were presented twice during the recognition phase; hence making the total number of trials in the recognition phase to be 110. In each trial, participants were instructed to make a judgment on whether a word was “old” or “new” by pressing a different button for each stimulus category. The words appeared on screen until the participant’s response or a maximum of 2000 ms and the interval between stimuli was jittered between 800 and 1200 ms.

Performance in the task was measured with the percentage of correct responses (i.e., answering “new” to a new word or answering “old” to an old word), and the reaction time to correct responses (Table 3).

### Resting-state electroencephalographic recording

Electroencephalographic activity was recorded throughout the experimental session between 0.001 and 100 Hz with a 50 Hz notch filter and digitized at 500 Hz using 60 Ag-AgCl active scalp electrodes positioned according to the 10–10 system (Chatrian et al., 1985) with nose tip reference and a ground electrode at Fp1. Furthermore, vertical and horizontal EOG were recorded with two electrodes positioned on the outer canthi of both eyes (HEOG) and two electrodes placed above and below the right eye (VEOG) to monitor blinking and eye movements. Electrode impedances were kept below 10 kΩ.

Resting-state electroencephalographic data was recorded at the end of the session for each participant, 5 min after finalizing the memory tasks. rsEEG was recorded for 3 min while the participants were sitting relaxed with eyes closed on a comfortable armchair inside a noise and light attenuated Faraday chamber. A researcher visually monitored the participants and the EEG recording in order to control for drowsiness, compliance with instructions, participants’ movements or any confounding events that could affect the EEG recording.

### Resting-state electroencephalographic preliminary analysis

Analyses were run offline using the Matlab toolbox EEGLab (Delorme and Makeig, 2004). As a first step for this analysis, a detrending of the slow fluctuation from the recorded scalp rsEEG traces was performed. Then, the 3 min of rsEEG data were divided into segments of 2 s. These segments were visually inspected in order to identify EEG segments that were affected by physiological (e.g., ocular, muscular, head movements…) or non-physiological (e.g., bad electrode contact, etc.) artifacts or that coincided with behavioral annotations taking during the recording (e.g., drowsiness, coughs, opened eyes, etc.). In this visual analysis, segments with behavioral artifacts and those that were deemed impossible to correct with posterior processing steps, as well as datasets that did not retain a minimum of 50 segments were discarded (final number of analyzed segments: CU–*x̄* = 75.63; *SD* = 8.57; range = 56–89; aMCI–*x̄* = 67.25; *SD* = 10.67; range = 50–88).

This was followed by an independent component analysis (ICA) decomposition and the removal of the estimated independent components showing residual instrumental or biological (i.e., ocular movements and/or EMG) artifacts (number of IC removed: CU–*x̄* = 7.83; *SD* = 2.72; range = 2–12; aMCI–*x̄* = 9.86; *SD* = 2.99; range = 5–16). EEGLab was used to reconstruct the signal without the artifactual components.

### Scalp power density of resting-state electroencephalographic rhythms

For each participant, a digital Fast Fourier Transform (FFT)–based power spectrum analysis computed the scalp power density of the normalized rsEEG signal averaged across all artifact–free segments, with a frequency resolution of 0.5 Hz at each electrode and frequency bin (0.5–45 Hz). After this, the global scalp normalized rsEEG power density was computed averaging the power density values across the 60 electrodes at each frequency bin. This global normalized rsEEG power density at the scalp level was used to obtain the frequency band values as follows:

The rsEEG frequency bands of interest were identified based on the transition frequency (TF) and the individual alpha frequency peak (IAFp) independently for each participant (Klimesch, 1999). The TF refers to the TF between the theta and alpha bands, and is defined as the minimum power density between 3 and 8 Hz; meanwhile, the IAFp is defined as the maximum power density peak between 6 and 14 Hz. Both landmarks were identified based on the average power density values across the 60 scalp electrodes. Specifically, as we were interested in posterior alpha activity, the alpha band range was calculated for each participant from the midpoint of the TF–IAFp range to IAFp +2 Hz. Thus, capturing the so-called alpha-2 (or lower-2 alpha) and alpha-3 (or upper alpha) sub-bands (Klimesch, 1999; Babiloni et al., 2020a).

After obtaining the individualized alpha band’s range, these ranges were applied to the estimation of the rsEEG cortical sources of each participant.

### Estimation of resting-state electroencephalographic cortical sources by exact low-resolution brain electromagnetic tomography

The topographical analysis of rsEEG rhythms in aMCI patients has been typically performed at scalp electrodes and may be affected by reference electrode and head volume conduction effects, which blur spatial analysis of the distribution of rsEEG potentials (Nunez and Cutillo, 1995). In the eyes closed condition, electrode reference and head volume conduction effects can bring neural ionic currents from anterior to posterior (location of the dominant alpha activity in normal individuals) cortical regions, so posterior scalp electrodes can capture neural ionic currents not generated in the posterior cerebral cortex. For this reason, the use of EEG source estimation is a useful methodological approach to investigate the dominant posterior alpha rhythms in EEG activity recorded in the resting state eyes-closed condition. Indeed, EEG source estimation is independent of the electrode reference effect and mitigates the head volume conduction effects (Schoffelen and Gross, 2009; Pascual-Marqui et al., 2011). To enhance such a spatial analysis, one can use analytical procedures to estimate cortical sources of eyes-closed rsEEG alpha rhythms. For example, a popular approach for the rsEEG cortical linear inverse source estimation is exact low-resolution brain electromagnetic tomography (eLORETA) (Pascual-Marqui, 2007). eLORETA uses a brain source space co-registered to the Talairach brain atlas often adopted in neuroimaging studies.

The freeware tool eLORETA was, thus, used to estimate the cortical source activation of the scalp–recorded EEG signal (Pascual-Marqui, 2002). The present implementation of eLORETA uses a head volume conductor model composed of the scalp, skull, and brain. In the scalp compartment, electrodes can be virtually positioned to give EEG data as an input to the source estimation (Pascual-Marqui, 2007). The brain model is based on a realistic cerebral shape taken from a template typically used in the neuroimaging studies, namely, that of the Montreal Neurological Institute (MNI152 template). The eLORETA freeware solves the so-called EEG inverse problem estimating “neural” current density values at any cortical voxel of the mentioned head volume conductor model. The solutions are computed for the rsEEG frequency bin-by-frequency bin.

The input for eLORETA source estimation was the artifact-free EEG epochs with 60 scalp electrodes placed according to the 10–10 montage system. This data was resampled to 256 Hz and filtered with a band pass filter between 0.5 and 45 Hz. From the resampled and filtered data, eLORETA solutions are computed at all rsEEG frequency bin-by-frequency bin (0.5 Hz as frequency resolution) by using the mean voltage values to calculate the cross–spectra matrix for each participant. These cross–spectra matrices are then feed to eLORETA algorithms to calculate the current source density estimates for 6239 voxels of 5 × 5 mm representing cortical gray matter and hippocampus, based on a 3-shell spherical head model registered to the Talairach brain atlas (Pascual-Marqui, 2002). For each voxel, the eLORETA package additionally provides the Talairach coordinates, the cortical lobe and the Brodmann area (BA).

The eLORETA estimates of neural current density were then normalized to reduce the effect of inter-subject variability in group analysis (Leuchter et al., 1993). The eLORETA solutions were normalized by the following procedure used in previous reference EEG studies by the present Workgroup (Babiloni et al., 2017a,b) (1) for the artifact-free rsEEG data of each participant, eLORETA computed its solution for all (6,239) cortical voxels and frequency bins (0.5–70 Hz); (2) the eLORETA solutions were averaged across all frequency bins from 0.5 to 45 Hz and all cortical voxels, to obtain the eLORETA “mean” solution; (3) for each frequency bin at each cortical voxel, we computed the ratio between the original eLORETA solution at that frequency bin and cortical voxel, and the eLORETA “mean” solution, in order to obtain the eLORETA “normalized” solution at that frequency bin and cortical voxel. This procedure was repeated for all frequency bins and cortical voxels.

After normalization, the current density values within the individualized alpha frequency band were averaged in order to obtain a single alpha frequency band value for each voxel. Further, a regional analysis of the eLORETA solutions was performed. Since, in the eyes closed condition, the alpha rhythms are dominant in the posterior cerebral cortex and given there may be a slight anteriorization of rsEEG alpha source activity in the progression from healthy aging to ADD (Osipova et al., 2006; Ishii et al., 2010), two macro-regions of interest (ROIs) comprising a single cerebral lobe were considered in order to better understand the behavior of alpha activity after the cognitive tasks and whether there are differences between CU and aMCI participants. The selected ROIs were the parietal lobe and the occipital lobe. The eLORETA solutions for each ROI were obtained by averaging the normalized eLORETA alpha current density values estimated at all single voxels included in BA 5, 7, 30, 39, 40, and 43 for the parietal ROI and those included in BA 17, 18, and 19 for the occipital ROI.

### Statistical analyses

All statistical analyses were performed by the commercial tool STATISTICA 10 (StatSoft Inc., Hamburg, Germany).^1^ Prior to the analyses all variables were screened for the presence of values deviating more than ±3 SD form the variable mean, as those values would be considered outliers. However, no outliers were detected in the analyzed variables.

The first statistical analysis was performed to correlate the relevant variables of tasks performance (i.e., cognitive engagement) with the parietal and occipital alpha power density across the CU and aMCI groups considered separately. The Pearson test was used for all the correlations between the EEG variables and task performance, as all variables had a Gaussian distribution (confirmed by the Kolmogorov–Smirnov test with a *p* > 0.05).

The second statistical analysis was performed in order to correlate the relevant neuropsychological scores with the parietal and occipital alpha power density in both CU and aMCI groups considered separately. Spearman test was used for the correlation of parietal and occipital alpha with MMSE scores, since the latter do not fit a normal distribution. As the rest of the variables had a normal distribution; thus, the Pearson test was used for the correlation of parietal and occipital alpha with the Trail Making Test Index and with the CAMCOG-R total score.

For both statistical analyses, in order to reduce the effect of false positive in statistical testing, the correlation coefficients were considered significant when: (a) their associated *p*-value was ≤0.05; and (b) the correlation coefficient represented at least moderate effect sizes following Cohen’s recommendations for interpreting correlation coefficients (i.e., *r* ≥ 0.3) (Cohen, 1988). We assumed this method in order to avoid overly conservative multiple comparisons control methods like the Bonferroni correction (Bender and Lange, 2001; Lee et al., 2021), given the exploratory nature of the present study. Additionally, despite not being considered as regards the significance of the correlation coefficients, 95% Confidence Intervals (CI) based on 5000 bootstrap permutations for all correlation coefficients are reported.

The third statistical analysis was performed in order to assess whether there were differences between diagnostic groups in the correlation coefficients of those correlations that were statistically significant at least for one group. To this end, a computation based on Fisher’s *r* to *Z* transformation (Fisher, 1921) was used in order to compare the correlation coefficients calculated for each group independently. The statistical test to compare both correlation coefficients is defined as:


(1) $$Z=\frac{Z_{1}-Z_{2}}{\sigma_{Z_{1}-Z_{2}}}$$

Where *Z*_1_ and *Z*_2_ are the Fisher *Z*-transformations of each group’s correlation coefficient, whereas σ_*Z*_1_-*Z*_2__ is the standard deviation, calculated as follows:


(2) $$\sigma_{Z_{1}-Z_{2}}=\sqrt{\sigma_{Z_{1}}^{2}+\sigma_{Z_{2}}^{2}}=\sqrt{\frac{1}{n_{1}-3}+\frac{1}{n_{2}-3}}$$

Where *n* is the sample size for each group.

Therefore, this test gives a *Z*-value that indicates whether the difference between the correlation coefficients of each group is statistically significant (i.e., *Z* statistic with an absolute value ≥ 1.96). This test was applied for all the variables where a significant correlation in at least one of the groups was observed.

## Results

### Resting-state electroencephalographic alpha exact low-resolution brain electromagnetic tomography source solutions in the cognitively unimpaired and amnestic mild cognitive impairment groups

Table 4 reports the mean values of TF and IAF for the CU and aMCI groups. A *t*-test for independent groups showed no differences between the CU and aMCI participants neither in TF, *t*-value = 0.053; *p* = 0.96, nor IAF, *t*-value = −0.661; *p* = 0.51 mean values (see Table 4).

**TABLE 4:** Mean values (±SD) of the transition frequency (TF) and individual alpha peak (IAF) computed from resting state EEG (rsEEG) eLORETA source solutions (normalized source current density) in the groups of cognitively unimpaired (CU) and amnestic mild cognitive impairment (aMCI) participants.

	CU	aMCI
*n*	30	40
TF (Hz)	5.67 (±1.16)	5.65 (±1.42)
IAF (Hz)	9.07 (±1.19)	9.26 (±1.25)

**FIGURE 1:**
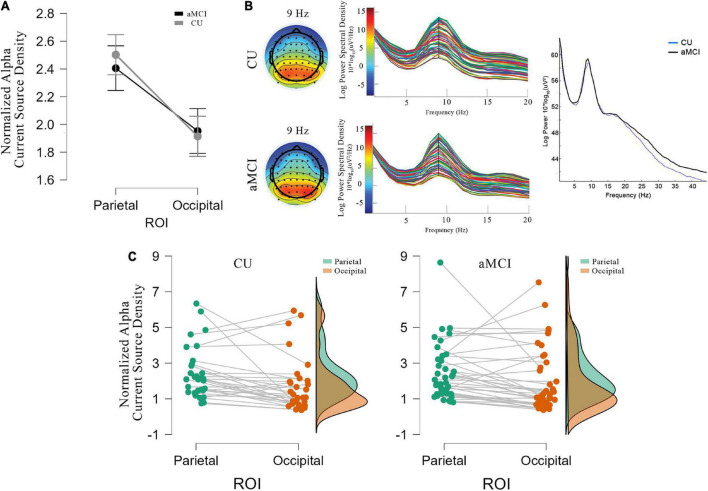
**(A)** Mean values and standard errors (denoted by the vertical bars) for the eLORETA source solutions (normalized alpha current source density) of rsEEG occipital and parietal alpha for CU (gray line) and aMCI (black line). **(B)** Topographic distribution of alpha activity at 9 Hz (closest to IAFp), and power spectra for each electrode for CU (top) and aMCI (bottom) groups, together with the mean power spectra between 0.5 and 45 Hz for both groups (CU in blue and aMCI in black). **(C)** Individual values of the regional normalized eLORETA source solutions (normalized alpha current source density) of rsEEG alpha in the parietal (green dots and area) and occipital (brown dots and area) regions of interest for CU (left) and aMCI (right) groups.

Figures 1A–C shows the mean values (±SE) of the rsEEG eLORETA source solutions (i.e., normalized current source density) for the alpha frequency band in the parietal and occipital ROIs in the CU and aMCI groups along with the individual values for the elements of each group. There was no significant ANOVA interaction effect among the factors Group (CU and aMCI) and ROI (parietal and occipital), *F*_(1,68)_ = 0.49; *p* = 0.487, nor significant differences between groups, *F*_(1,68)_ = 0.04; *p* = 0.843 in the values of normalized alpha current source density.

### Correlation between resting-state electroencephalographic posterior normalized alpha current density and cognitive engagement

Tables 5, 6 report correlation coefficients computed between the performance variables and the mean normalized alpha current density in the parietal and occipital ROIs for each group.

**TABLE 5:** Correlation coefficients (Pearson’s *r*) and correspondent 95% CI between eLORETA source solutions (normalized alpha current source density) of parietal and occipital alpha rsEEG rhythms and episodic memory task performance for CU seniors and patients with aMCI.

	Parietal alpha				Occipital alpha			
	CU		aMCI		CU		aMCI	
Correct responses (%)	−0.193	[−0.57, 0.22]	0.206	[−0.02, 0.41]	−0.107	[−0.46, 0.52]	0.111	[−0.18, 0.34]
Mean reaction time to correct responses (ms)	0.436*	[0.08, 0.69]	−0.094	[−0.37, 0.17]	0.108	[−0.51, 0.43]	−0.121	[−0.40, 0.23]

A good performance in the task is indicated by high % of correct answers and low reaction times. Significant values with *p* < 0.05 are marked in bold.

**TABLE 6:** Correlation coefficients (Pearson’s *r*) and correspondent 95% CI between eLORETA source solutions (normalized alpha current source density) of parietal and occipital alpha rsEEG rhythms and working memory task performance for CU and aMCI subjects.

	Parietal alpha				Occipital alpha			
	CU		aMCI		CU		aMCI	
Correct responses (%)	−0.359*	[−0.67, 0.12]	−0.001	[−0.55, 0.09]	0.087	[−0.34, 0.44]	−0.110	[−0.49, 0.21]
Mean reaction time to correct responses (ms)	0.242	[−0.11, 0.54]	0.098	[−0.17, 0.35]	0.348	[−0.01, 0.69]	0.002	[−0.27, 0.34]
Mean reaction time to errors (ms)	0.352	[0.03, 0.60]	0.069	[−0.24, 0.35]	0.415*	[0.08, 0.73]	−0.045	[−0.35, 0.33]

A good performance in the task is defined by a high % of correct answers and low reaction times. Significant values with *p* < 0.05 are marked in bold.

**FIGURE 2:**
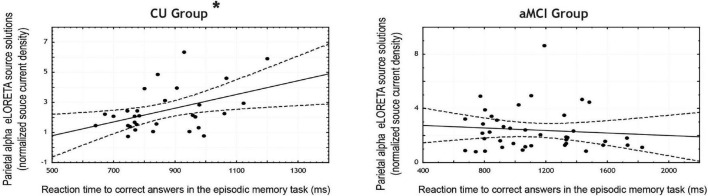
Scatterplots for the CU and aMCI groups showing the correlation between eLORETA source solutions (normalized alpha current source density) of parietal alpha resting state EEG (rsEEG) rhythms and the reaction time to correct answers in the episodic memory task (in ms). Note that * indicates significant correlations (*p* < 0.05 and *r* > 0.3).

In the CU seniors, there was a statistically significant positive correlation between the normalized alpha current density in the parietal ROI and the mean reaction time to correct responses in the EM task. The higher the parietal normalized alpha current density, the longer the reaction time in correct trials for both types of words. No statistically significant correlations were found for the aMCI group, nor in the occipital ROI for both groups (see Table 5 and Figure 2).

**FIGURE 3:**
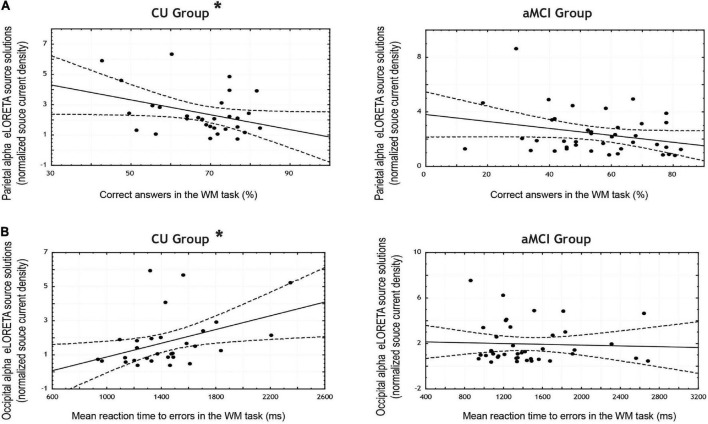
Scatterplots for the CU and aMCI groups showing the correlation between eLORETA source solutions (normalized alpha current source density) of: **(A)** parietal alpha and the percentage of correct answers in the working memory task; and **(B)** occipital alpha and the mean reaction time to errors in the working memory task (in ms). Note that * indicates significant correlations (*p* < 0.05 and *r* > 0.3).

Regarding the WM task, in the CU seniors there was a statistically significant negative correlation between the normalized alpha current density in the parietal ROI and the percentage of correct responses (note that the 95% CI points to marginal significance). The higher the normalized alpha current density, the lesser number of correct responses in the WM task. Furthermore, the CU seniors also showed a statistically significant positive correlation between the normalized alpha current density in the occipital ROI and the mean reaction time to errors; the higher the normalized alpha current density, the longer the reaction time when making an error. No statistically significant correlations were found in the aMCI group (see Table 6 and Figures 3A,B).

### Correlation between resting-state electroencephalographic posterior normalized alpha current density and global cognitive function (neuropsychological scores)

**FIGURE 4:**
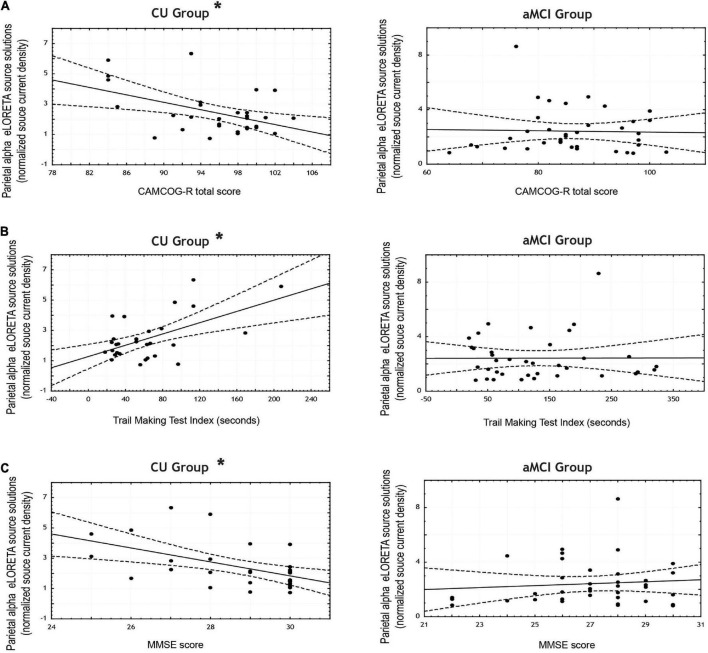
Scatterplots for the CU and aMCI groups showing the correlations between eLORETA source solutions (normalized alpha current source density) of parietal alpha resting state EEG (rsEEG) rhythms and the scores in the neuropsychological tests: **(A)** CAMCOG total score; **(B)** Trail Making Test Index (in seconds); and **(C)** MMSE score. Note that * indicates significant correlations (*p* < 0.05 and *r* > 0.3).

Table 7 reports the correlation coefficients computed between the scores obtained in the neuropsychological tests and the mean normalized alpha current density in both the parietal and occipital ROIs for each group (see Figures 4A–C for the scatterplots).

**TABLE 7:** Correlation coefficients (Pearson’s *r*) and correspondent 95% CI between eLORETA source solutions (normalized alpha current source density) of parietal and occipital alpha rsEEG rhythms and neuropsychological test scores for CU and aMCI groups.

	Parietal alpha				Occipital alpha			
	CU		aMCI		CU		aMCI	
CAMCOG Battery	−0.478**	[−0.75, −0.04]	−0.029	[−0.29, 0.34]	−0.205	[−0.63, 0.38]	0.108	[−0.23, 0.40]
TMT Derived Index (B-A)	0.565**	[0.13, 0.78]	−0.104	[−0.35, 0.29]	0.013	[−0.32, 0.42]	0.004	[−0.37, 0.27]
MMSE	−0.494**^a^	[−0.73, −0.17]	0.078	[−0.26, 0.48]	−0.237*^a^*	[−0.55, 0.16]	0.013	[−0.27, 0.42]

MMSE, mini-mental state evaluation; CAMCOG, cambridge cognition examination.

^a^Spearman’s *r* used due to data not fitting a normal distribution.

**p < 0.01. Note that for the Trail Making Test Index a better cognitive function is indicated by a lower positive value. For the rest of tests, a higher value indicates a better cognitive function.

Significant values with *p* < 0.05 are marked in bold.

In the CU seniors there were statistically significant negative correlations between the normalized alpha current density in the parietal ROI and the MMSE score, and the CAMCOG battery total score; as well as a statistically significant positive correlation between the normalized alpha current density in the parietal ROI and the TMT index. That is, the higher the parietal normalized alpha current density, the lower the MMSE and CAMCOG scores, and the higher the TMT index.

### Differences between amnestic mild cognitive impairment and cognitively unimpaired correlation coefficients

Table 8 reports the correlation coefficients that were compared between groups for the cognitive tasks and parietal/occipital normalized alpha current density; and for the neuropsychological scores with parietal normalized alpha current density, alongside the *p*-values resulting from their comparison. Note that only correlation coefficients that yielded a significant correlation for at least one group were compared.

**TABLE 8:** Correlations (Pearson’s *r*) between eLORETA source solutions (normalized source current density) of parietal and occipital alpha rsEEG rhythms and episodic memory task, working memory task, and neuropsychological test scores for CU and aMCI groups, alongside *p*-values of the statistical comparison between the correlation coefficients of each group for each correlation.

Correlation	CU	aMCI	*Z*-value	*P*-value
**Episodic memory task**				
Mean reaction time to correct responses (ms) with Parietal alpha activity	0.436	−0.094	2.22	**0.03**
**Working memory task**				
Correct responses (%) with Parietal alpha activity	−0.359	−0.001	−0.35	0.70
Mean reaction time to errors (ms) with Occipital alpha activity	0.415	−0.045	1.18	0.24
**Neuropsychological data**				
CAMCOG-R total score with Parietal alpha activity	−0.478	−0.029	−1.94	**0.05**
TMT derived index with Parietal alpha activity	−0.565	0.104	−2.54	**0.01**
MMSE with Parietal alpha activity	−0.494*^a^*	0.078*^a^*	−2.45	**0.01**

^a^Spearman’s r used due to data not fitting a normal distribution.

Significant values with *p* < 0.05 are marked in bold.

In the EM task, statistically significant differences were found between CU and aMCI seniors in the correlation coefficients between parietal alpha activity and the mean reaction time for correct responses in the task, with CU participants showing greater coefficients than aMCI seniors.

However, in the WM task no significant differences were found between CU and aMCI seniors in the correlation coefficients between neither parietal alpha activity and accuracy nor occipital alpha activity and the mean reaction time to errors in the task.

Lastly, in the neuropsychological scores, significant differences were found between CU and aMCI in the correlation coefficients between parietal alpha activity and the total score of the CAMCOG-R battery, the MMSE score, and the TMT index; in all three cases, CU participants showed a stronger correlation between the two variables than aMCI seniors.

## Discussion

In the present retrospective and exploratory study, the archived rsEEG data of CU and aMCI seniors were analyzed. The rsEEG data were recorded 5 min after the performance of two memory tasks. It was explored if background rsEEG alpha activity after such a short interval at the end of cognitive tasks execution show similar properties to rsEEG alpha recorded at basal conditions in CU and aMCI participants. The exploratory nature of this study is due to the lack of rsEEG recordings before the cognitive tasks and after several time intervals between those tasks and the rsEEG recordings.

### The cognitively unimpaired (but not amnestic mild cognitive impairment) participant’s cognitive engagement in the memory tasks is related to posterior resting-state electroencephalographic alpha activity

A main result of the present study showed that the CU (but not aMCI) participants’ memory tasks performance (i.e., reaction time and accuracy during about 40 min of memory demands) were negatively related to the posterior rsEEG alpha activity recorded 5 min after the end of those tasks. The better the memory performance during those tasks, the lower the posterior rsEEG alpha activity. Specifically, there was a significant positive correlation between reaction time for correct responses in the EM task and parietal alpha activity. Additionally, there was a significant negative correlation between percentage of correct responses in the WM task and parietal alpha activity, as well as a significant positive correlation between reaction time for errors in the WM task and occipital alpha activity. Notably, the interpretation of this finding should be done in light of the well-known fact that low posterior rsEEG alpha activity reflects high cortical arousal.

At basal conditions, rsEEG alpha activity has been correlated with performance in different cognitive tasks (Mahjoory et al., 2019; Clements et al., 2021) but generally in the opposite direction: i.e., a greater alpha power at rest tends to correlate with better task performance. This discrepancy could indicate that rsEEG alpha activity in the present study is affected by the task performance itself while it is not in basal conditions studies since rsEEG alpha activity is recorded before the task presentation (Clements et al., 2021) or in different days (Mahjoory et al., 2019). Thus, recording rsEEG alpha activity minutes after performing the memory tasks, could have led to alpha activity at rest being affected by the task. For instance, during visual WM tasks, alpha activity has been found to be typically reduced over occipital task-relevant regions and has been related to task performance (van Ede et al., 2017; van Ede, 2018), as well as over parietal regions during EM old/new memory tasks (Hanslmayr et al., 2012); both type of tasks being similar to the ones used in this study. This reduction of event-related alpha activity has been suggested to reflect the disinhibition of relevant cortical networks, facilitating neural communication and task performance (Hanslmayr et al., 2016; Griffiths et al., 2019) and would be located over cortical regions relevant to the tasks (van Ede et al., 2017). This framework could explain the correlations with parietal and occipital alpha power at rest in the present work. The fact that significant correlations between performance and parietal alpha activity at rest were found in both tasks, independently of stimulus (images vs. words) or memory modality (working vs. episodic), indicates that both tasks would need to successfully engage attentional processing, which has been linked to parietal regions (Raz, 2004; Raz and Buhle, 2006).

Following that logic, it can be speculated that the participants’ brain activity in the memory systems during those tasks still affected the cortical arousal in quiet vigilance, as revealed by posterior rsEEG alpha activity 5 min after the intense memory efforts. Such a short period may not allow a recovery of brain arousal to basal levels as those typically observed preceding the memory tasks. Given the inverse correlation between memory tasks performance and the subsequent posterior rsEEG alpha activity in the present CU persons, it can be also speculated that better performance in the memory tasks may reflect greater engagement of brain memory systems and greater task-related cortical arousal prolonged during the time of rsEEG recordings in the present experimental conditions. However, this should be taken with caution as it is only one of a number of possible alternative explanations. For instance, it could also be that the current sample of CU participants have a generally low alpha level and that they have already recovered they low alpha level after task engagement or that they have never engaged in the task and maintain their low alpha level across the experimental session. Albeit these alternatives may be less plausible given the participants’ performance level in the task and the correlation analyses results, they are still plausible. Hence, to add support to the presented explanation and rule out the aforementioned alternative explanations, future works are needed studying resting state alpha activity before and after the performance of different cognitive tasks.

### The cognitively unimpaired (but not amnestic mild cognitive impairment) participant’s global cognitive function is related to posterior resting-state electroencephalographic alpha activity

Another interesting result of this study was that the CU (but not aMCI) participants’ global cognitive function, as measured by the scores of standard neuropsychological tests (e.g., MMSE, CAMCOG Battery, and TMT), was negatively related to the magnitude of posterior rsEEG alpha activity recorded 5 min after the end of the memory tasks. The better the global cognitive function, the lower the posterior rsEEG alpha activity in the CU participants.

This result appears to be in disagreement with previous evidence repeatedly showing that posterior rsEEG alpha activity recorded at basal conditions was positively correlated to global cognitive function in the elderly (Ishii et al., 2018; Babiloni et al., 2020b). Specifically, parietal rsEEG alpha activity was positively correlated with global cognitive function as measured by neuropsychological tests, namely the better the cognitive functioning as revealed by CAMCOG or MMSE scores, the higher the posterior rsEEG alpha activity in CU persons and ADD patients with cognitive deficits (Claus et al., 2000; Babiloni et al., 2011, 2013, 2014, 2015, 2018; Musaeus et al., 2018).

However, current results do not necessarily contradict the previous literature. In this case, given the circumstances in which the rsEEG was recorded (right after two memory tasks) it may be possible that the relationship between alpha activity and global cognitive function is affected in the same way as posterior alpha activity was affected by previous cognitive engagement when relating it to the preceding tasks performance. Therefore, it can be speculated that those persons with better global cognitive function may be more involved in the memory tasks with their cortical activation affecting the cortical arousal in quiet vigilance more intensively than that of those with worse global cognitive function, as revealed by posterior rsEEG alpha activity 5 min after the intense memory efforts. Nonetheless, this remains a possibility among other plausible explanations. Hence, besides taking the present interpretation with caution, future studies on the recovery (see Supplementary Table 1 for a tentative exploratory analyses) and relationship of resting state alpha activity after the performance of cognitive tasks with the individual global cognitive status are needed to add support to the proposed explanation.

### Differences between amnestic mild cognitive impairment and cognitively unimpaired and influence of cognitive status

It was found that the magnitude of the significant correlations between posterior alpha activity and task performance, as well as the significant correlations between parietal alpha activity and global cognitive function depended on the cognitive status (i.e., CU vs. aMCI diagnosis) of the participants. Namely, it was found that the correlations between lower parietal alpha activity and greater EM performance and global cognitive function were stronger in the CU group compared to the same correlations in the aMCI group. Furthermore, these correlations were only significant in the CU group.

In this light, it can be speculated that aMCI participants may have less cognitive engagement while performing the task. In fact, many aMCI patients show frontal executive dysfunctions (Brandt et al., 2009; Johns et al., 2012; Zheng et al., 2012, 2014; Zurron et al., 2018; Jung et al., 2020) that may prevent their cognitive engagement while performing the memory tasks, which, in turn, may affect the subsequent rsEEG recording in the present aMCI patients. Therefore, if rsEEG alpha activity recorder after the execution of cognitive tasks reflects the level of engagement in those tasks, it could be the case that the present results showed a milder engagement of aMCI than CU participants in those tasks. That is, CU individuals may show a low alpha level due to their engagement in the preceding cognitive tasks, while aMCI individuals may present their usually low alpha level (i.e., lower than healthy controls in resting state studies) due to their lack of engagement with the tasks. This fact, could, in turn, obscure between groups differences in rsEEG alpha activity in the present work, precluding as to observe the well-established reduction of resting state alpha activity in aMCI when compared with healthy controls (reviewed in Lejko et al., 2020). Nonetheless, as for the previous results, future studies that allow assessing whether aMCI may have either recovered their background basal alpha activity levels at the time of the recording after task execution or whether they had not had alpha modulated during task execution due to their lower engagement are needed to further deepen on the topics opened by the present results.

### Limitations

Since this is a retrospective study, the biggest limitation that was faced was the data available to analyze, as there was no available rsEEG recordings previous to nor during the memory task in order to properly compare the posterior alpha activity before and after the memory tasks or the levels of alpha activity during cognitive engagement. In this sense, further studies about this topic should not only record activity at rest before and after tasks, but also with different time intervals after the tasks end (e.g., 5, 15, and 30 min), in order to narrow down the right time interval between tasks where baseline brain oscillatory activity is recovered.

## Conclusion

Background cortical arousal as reflected by alpha activity recorded a few minutes after a cognitive task showed different properties and relationships with cognition than those reported for alpha activity when recorded at basal conditions. More importantly, it is suggested that this effect on background brain activity or its duration might be different depending on the participant’s cognitive status, which should be taken into account when comparing groups with varied degrees of cognitive health (e.g., healthy seniors and aMCIs) with blocks of successive cognitive tasks, as results obtained might be affected by a differential recovery of background oscillatory activity.

## Data availability statement

The data analyzed in this study is subject to the following licenses/restrictions: The present study was developed based on the data of the informal European Consortium PDWAVES. EEG datasets analyzed were part of the Spanish datasets collected in said Consortium and have not been made publicly available. Requests to access these datasets should be directed to www.pdwaves.eu.

## Ethics statement

The studies involving human participants were reviewed and approved by Galician Clinical Research Ethics Committee (Xunta de Galicia, Spain). The patients/participants provided their written informed consent to participate in this study.

## Author contributions

AF performed the EEG data processing and statistical analysis and wrote the first draft of the manuscript. GN, CD, and CB supervised the EEG data processing and statistical analysis, contributed to the interpretation of the results, and revised the manuscript. DP helped with statistical analysis and wrote the manuscript. FD obtained funding, supervised the study, and revised the manuscript. CL-S provided the neuropsychological data and diagnosis and helped to write the diagnosis criteria in the manuscript. All authors contributed to the article and approved the submitted version.

## Footnotes

1
www.statsoft.com
