# Supplementary material for: Resting state electroencephalographic rhythms are affected by immediately preceding memory demands in cognitively unimpaired elderly and patients with mild cognitive impairment
Source: Front Aging Neurosci. 2022 Aug 5;14:907130. doi: 10.3389/fnagi.2022.907130 (PMC9435320; doi:10.3389/fnagi.2022.907130)
Supplement: Supplementary file 1 [file Table_1.DOCX]

Supplementary Material

# Supplementary Tables

A mixed repeated measures ANOVA with the between participants factor Group (CU and aMCI) and the within participants factor Minute (1st, 2nd and 3rd) and ROI (Parietal and Occipital) was run on an estimation of normalized alpha current source density for each single minute of the resting state period, trying to shed light into the dynamics of alpha activity. Results from these analyses showed that there were no statistically significant main effects of Minute, *F*(1.575, 102.390) = 0.525, *p* = 0.550; nor Group, *F*(1, 65) = 0.354, *p* = 0.554;. Also, there were no significant interactions between Minute and Group, *F*(1.575, 102.390) = 2.448, *p* = 0.104; nor the triple interaction Minute by Group by ROI, *F*(1.374, 89.311) = 0.457, *p* = 0.562. All in all, dynamics of normalized alpha current source density do not show an identifiable pattern across the three minutes of resting state, and albeit CU generally showed lower normalized alpha current source density than aMCI there are no statistical differences between both groups. These analyses are to be taken with caution since the number of segments averaged for each minute is not enough as to offer a reliable estimate of rsEEG alpha dynamics.

## Supplementary Table 1

**Supplementary Table 1.** CU and aMCI mean normalized alpha current source density (± SD) for each ROI for every minute of the resting state period.

|  | **Parietal ROI** | | | **Occipital ROI** | | |
| --- | --- | --- | --- | --- | --- | --- |
|  | *1^st^ minute* | *2^nd^ minute* | *3^rd^ minute* | *1^st^ minute* | *2^nd^ minute* | *3^rd^ minute* |
| **CU** | 2.84 (± 1.72) | 2.94 (± 1.84) | 2.57 (± 1.59) | 2.11 (± 1.86) | 2.19 (± 1.86) | 1.89 (± 1.68) |
| **aMCI** | 2.90 (± 1.96) | 2.86 (± 2.04) | 2.96 (± 1.99) | 2.60 (± 3.54) | 2.33 (± 2.20) | 2.57 (± 2.74) |
| Note that the number of 2-second segments used for the estimation of normalized alpha current source density at each minute within the resting state period is low. Hence, caution is required to interpret values of these estimations since they may not be robust or reliable enough. | | | | | | |
